# TRPC1 mediates slow excitatory synaptic transmission in hippocampal *oriens/alveus* interneurons

**DOI:** 10.1186/s13041-020-0558-9

**Published:** 2020-01-29

**Authors:** André Kougioumoutzakis, Joe Guillaume Pelletier, Isabel Laplante, Abdessattar Khlaifia, Jean-Claude Lacaille

**Affiliations:** Department of Neurosciences and GRSNC, Université de Montréal, P.O. Box 6128, Station Downtown, Montreal, Montreal, QC H3C 3J7 Canada

**Keywords:** Hippocampus, GABA interneurons, TRPC subtypes, siRNA, mGluR1a-mediated slow EPSC

## Abstract

Hippocampal GABAergic interneurons play key roles in regulating principal cell activity and plasticity. Interneurons located in stratum *oriens/alveus* (O/A INs) receive excitatory inputs from CA1 pyramidal cells and express a Hebbian form of long-term potentiation (LTP) at their excitatory input synapses. This LTP requires the activation of metabotropic glutamate receptors 1a (mGluR1a) and Ca^2+^ entry via *transient receptor potential* (TRP) channels. However, the type of TRP channels involved in synaptic transmission at these synapses remains largely unknown. Using patch-clamp recordings, we show that slow excitatory postsynaptic currents (EPSCs) evoked in O/A INs are dependent on TRP channels but may be independent of phospholipase C. Using reverse transcription polymerase chain reaction (RT-PCR) we found that mRNA for TRPC 1, 3–7 was present in CA1 hippocampus. Using single-cell RT-PCR, we found expression of mRNA for TRPC 1, 4–7, but not TRPC3, in O/A INs. Using co-immunoprecipitation assays in HEK-293 cell expression system, we found that TRPC1 and TRPC4 interacted with mGluR1a. Co-immunoprecipitation in hippocampus showed that TRPC1 interacted with mGluR1a. Using immunofluorescence, we found that TRPC1 co-localized with mGluR1a in O/A IN dendrites, whereas TRPC4 localization appeared limited to O/A IN cell body. Down-regulation of TRPC1, but not TRPC4, expression in O/A INs using small interfering RNAs prevented slow EPSCs, suggesting that TRPC1 is an obligatory TRPC subunit for these EPSCs. Our findings uncover a functional role of TRPC1 in mGluR1a-mediated slow excitatory synaptic transmission onto O/A INs that could be involved in Hebbian LTP at these synapses.

## Introduction

Hippocampal GABAergic interneurons represent a diverse population of inhibitory cells that are involved in gating information flow and computations by controlling principal cells activity [1, 2]. Interneuron functions are not static and inhibitory cells express short- and long-term plasticity of their synaptic inputs and outputs [3, 4]. Long-lasting changes at interneurons synapses may serve to enhance hippocampal network computation and flexibility [3, 5]. O/A INs are dendrite-projecting interneurons consisting mostly of oriens/lacunosum-moleculare (O-LM) cells, but also projection cells with additional subicular, retro-hippocampal or septal projections, as well as bistratified cells [2, 6]. O/A INs receive excitatory glutamatergic inputs from CA1 pyramidal cells that express a Hebbian form of LTP [5, 7, 8]. This LTP depends on the activation of metabotropic glutamate receptor subtype 1a (mGluR1a) and postsynaptic calcium elevation [9, 10]. At the circuit level, LTP of O/A IN input synapses regulates metaplasticity of CA1 pyramidal cells [5] and hippocampal long-term memory consolidation [11] .

High frequency stimulation of excitatory inputs onto O/A INs elicit slow excitatory postsynaptic currents (EPSCs) that are mediated by mGluR1a and non-selective cationic channels of the *transient receptor potential* (TRP) family [12]. Moreover, Ca^2+^ influx via TRP channels following mGluR1a activation is necessary for LTP induction at O/A IN excitatory synapses [13]. However, which TRP channel is involved in transmission and plasticity at these synapses remains unknown.

The canonical TRP (TRPC) subfamily contains seven members (TRPC1 to TRPC7) that form non-selective cation channels. TRPCs are highly expressed in brain including the cerebral cortex, hippocampus, cerebellum, and amygdala [14–18]. These channels play important role in neuronal development [19], synaptic plasticity [20] and memory [21, 22]. Deficits in TRPC function have been suggested to contribute to brain disorders like autism spectrum disorders, intellectual disability and bipolar disorder [23]. In the hippocampus, TRPC can be activated by different G-protein coupled receptors like tyrosine kinase receptors, mGluR1/5 and muscarinic cholinergic receptors usually through phospholipase C pathway engagement [24–27].

A number of studies have reported the involvement of TRPCs in mGluR1a-mediated synaptic transmission [28–33]. TRPC1 was reported to physically associate with mGluR1a receptors in the cerebellum and dopaminergic neurons of the substantia nigra (SN) [29, 34]. However other reports suggest also an implication of TRPC3 in cerebellar mGluR1a-mediated slow transmission [32, 35]. The presence of TRPCs is well documented in the hippocampus [16–18, 36–41], but their role in inhibitory interneurons remains largely unaddressed.

In this work, we examined in more detail the role of TRPCs in excitatory synaptic transmission in O/A INs. We found that O/A INs express mRNA for TRPC1, 4–7, and that TRPC1 was co-localized with mGluR1a in O/A IN dendrites. Moreover, siRNA knock-down of TRPC1, but not TRPC4, impaired slow EPSCs in O/A INs. Thus, TRPC1 is a necessary component of mGluR1a-mediated slow excitatory synaptic transmission in O/A INs, uncovering a possible role in induction of Hebbian LTP at these synapses.

## Materials and methods

### Acute hippocampal slices

Hippocampal acute slices were prepared from 3 to 4 weeks old Sprague-Dawley rats. Animals were anesthetized with isoflurane inhalation and the brain was rapidly removed and placed in ice-cold sucrose-based cutting solution containing (in mM): 250 sucrose, 2 KCl, 1.25 NaH_2_PO_4_, 7 MgSO_4_, 0.5 CaCl_2_, 26 NaHCO_3_, 10 glucose, pH 7.4, and 300 mOsmol/L. A block of tissue containing the hippocampus was prepared and 300 μm transverse hippocampal slices were obtained with a Leica VT1000S vibratome. Slices were transferred for recovery for 1 h at room temperature in artificial cerebral spinal fluid (ACSF) containing (in mM): 124 NaCl, 2.5 KCl, 1.25 NaH_2_PO_4_, 1.3 MgSO_4_, 2.5 CaCl_2_, 26 NaHCO_3_, and 10 glucose (pH 7.3–7.4, 295–305 mOsmol/L). Both cutting solution and ACSF were saturated with 95% O_2_/5% CO_2_.

### Organotypic hippocampal slice culture and biolistic transfection

Organotypic hippocampal slice cultures were prepared from 7 to 12 days old rats and biolistically co-transfected with a plasmid for EYFP expression and siRNAs as previously described [42]. Slices were transfected after 3 days in vitro and were used for experiments 2 days later. All small interfering RNAs (siRNAs) were purchased from Dharmacon (Lafayette, CO, USA). Commercial siControl was used as non-targeting control siRNA. TRPC1 siRNA was a combination of four target sequences whereas TRPC4 was a single sequence. All sequences are listed in Table 1.

**Table 1:** Small interfering RNA sequences

Target	Sense (5′ to 3′)	Antisense (5′ to 3′)
TRPC1	GAACAUAAAUUGCGUAGAUUU	PAUCUACGCAAUUUAUGUUCUU
	GGACUACGGUUGUCAGAAAUU	PUUUCUGACAACCGUAGUCCUU
	GAAUUUAAGUCGUCUGAAAUU	PUUUCAGACGACUUAAAUUCUU
	UGAACUUAGUGCUGACUUAUU	PUAAGUCAGCACUAAGUUCAUU
TRPC4	GCCAUUAAGUACCGUCAAAUU	PUUUGACGGUACUUAAUGGCUU

### Whole-cell recordings

Hippocampal slices were perfused continuously with ACSF (2.5 ml min^−1^, 31–33 °C). CA1 interneurons located in stratum oriens/alveus were visually identified using an upright microscope (Nikon Eclipse, E600FN) equipped with a water-immersion long-working distance objective (40x, numerical aperture 0.8) and an infrared video camera (70 Series, DAGE-MTI, MI, USA). EYFP-expressing cells were identified using an X-cite 120 arc lamp (EXFO photonic solution Inc., Mississauga, ON, Canada). Whole-cell voltage-clamp recordings were obtained using borosilicate glass pipettes (4–5 MΩ, WPI, Inc., FL, USA) filled with intracellular solution containing (in mM): 130 CsMeSO_3_, 5 CsCl, 2 MgCl_2_, 10 HEPES, 10 EGTA, 5 Na_2_- phosphocreatine, 2 ATP-Tris, 0.4 GTP-Tris, 1 QX314, pH 7.2–7.3, and 280 ± 5 mOsmol. Data was acquired using a Multiclamp 700A amplifier (Molecular Devices, CA, USA) and digitized using Digidata 1440A and pClamp 10 (Molecular Devices). Recordings were low-pass filtered at 2 kHz and digitized at 10 kHz. Series resistance was regularly monitored during experiments and data were included only if the holding current and series resistance were stable. To overcome potential biological variability and confirm experimental validity, in acute slice experiments, recordings were routinely obtained from only one cell per slice and repeated at least in three animals. For cultured slice experiments, recordings were obtained from only one cell per slice and from at least three independent culture experiments.

### Pharmacology

To isolate mGluR1a-mediated excitatory postsynaptic currents (mGluR1a-EPSCs), antagonists of non-NMDA (6-cyano-7-nitroquinoxaline-2,3-dione; CNQX, 20 μM), NMDA (DL-2-amino-5-phosphonopentanoic acid; AP5, 50 μM), and GABA-A (gabazine, 5 μM) receptors (all from Sigma, Oakville, ON, Canada), as well as the glutamate transporter blocker DL-*threo-b*-benzyloxyaspartic acid (TBOA, 30 μM) (Tocris, Ellisville, MO,USA) were bath-applied. In some experiments, the mGluR1a receptor antagonist (*S*)(+)-α-amino-4-carboxy-2-methylbenzeneacetic acid (LY367385, 100 μM), the TRP channel antagonist 1–2-(4-methoxyphenyl)-2-[3-(4-methoxyphenyl)proproxy]ethyl-1H-imidazole (SKF96365, 30 μM) (both drugs from Tocris), or the phospholipase C inhibitor 1-[6-[[(17β)-3-methoxyestra-1,3,5(10)-trien-17-yl]amino]hexyl]-1*H*-pyrrole-2,5-dione (U73122, 10 μM) (Calbiochem, Gibbstown, NJ, USA), were added to the external solution.

### RT-PCR

Rat brains were removed and dissected in Hank’s balanced salt solution (HBSS; Invitrogen, Carlsbad, CA, USA). Cortico-hippocampal slices (600 μm thick) were obtained using a McIlwain tissue chopper (Campen Instruments, Lafayette, IN, USA) and CA1 region was surgically isolated under a dissecting microscope. Total RNA was extracted from 20 mg of CA1 tissue with a GenElute Mammalian Total RNA Miniprep kit (Sigma). Reverse transcription was performed with 5 μg of CA1 extracted RNA using M-MLV reverse transcriptase (Invitrogen) following manufacturer’s protocol. All procedures were performed under RNase free conditions. For each member of the TRPC family, cDNA was amplified by PCR in separate reactions. Two microliters of the reverse transcription product were mixed with 0.1 μM of each primer, 1 U of *Taq* DNA polymerase (NEB), 0.1 mM of each dNTP, and 2 mM MgSO_4_ and amplified for 35 cycles using the following program: 93 °C, 30 s; 55 °C, 30 s; 72 °C, 45 s. Sequences of PCR primers and expected product sizes are listed in Table 2. Amplification of genomic DNA was excluded by intron-overspanning location of all primers.

**Table 2:** PCR primers sequences and expected product size

mRNA	Accessionnumber	Sense primer	Antisense primer	Product size
TRPC1	NM_053558	5′-AAGAGCAGAAG	5′-GTGCTCTGCATC	562 bp
		GACTGCGTAG-3′	TTCTGTCG-3′	
TRPC2	NM_022638	5′-CCTTCGAGTCAT	5′-CCTTGGTCTCCA	449 bp
		CAAGGCTC-3′	GATCTTCC-3′	
TRPC3	NM_021771	5′-CATCTTCATTGC	5′-CCACTCTACATC	622 bp
		TGCCTTCA-3’	ACTGTCATCC-3’	
		5′-CCTGAGCGAAGT	5′-CCACTCTACATC	529 bp
		CACACTCCCAC-3′^§^	ACTGTCATCC-3’	
TRPC4	NM_080396	5-ATGAGGAATCTG	5′-TATGTCTCTCGG	316 bp
		GTGAAGCG-3’	AGGCAATG-3’	
TRPC5	NM_080898	5′-C(G,T)ATGTTTGG	5′-TGCAGCCACATA	374 bp
		GACATACAACG-3’	TCT(C,T)TTGAC-3’	
TRPC6	NM_053559	5′-GTGCCAAGTCCA	5′-CTGGGCCTGCAG	314 bp
		AAGTCCCTGC-3’	TACGTATC-3’	
TRPC7	XM_225159	5′-CCTGTACTCCTA	5′-TGGTGACATTAT	182 bp
		CTACCGAGGTGC-3’	AAACGCCGTAC-3’	
GFAP	NM_017009	5′-ACCTCTGCACGC	5′-TCCAGCGACTCA	557 bp
		CGCTCCTATG-3’	ACCTTCCTCT-3’	
		5′-ATTCCGCGCCTC	5′-TTCATCCGCCTC	435 bp
		TCCCTGTCTC-3′^§§^	CTGTCTGT-3′^§§^	

^§^: Semi-nested PCR primer

^§§^: Nested PCR primer

### Multiplex single-cell RT-PCR

After whole-cell patch-clamp recording in acute slices, O/A interneuron cytoplasm was removed by applying gentle negative pressure to the patch pipette for 10 min. After withdrawal of the pipette from the slice, pipette solution (5–6 μl) was expelled into a microtube containing the reverse transcriptase mixture: 20 U RNase OUT (Invitrogen) and 10 mM DTT (12 μl final volume). Reverse transcription procedure was carried out as described above. cDNAs were then amplified by a two-step PCR. For the first round PCR, the whole reverse transcription product was separated in 2 tubes: the first one containing primers for TRPC1, 4, 5, 7 and the other containing primers for TRPC3, 6 and glial fibrillary acidic protein (GFAP). Each tube contained 0.1 μM of each primers, 2.5 U of *taq* DNA polymerase, 2 mM MgSO_4_ and no dNTP were added (50 μL of final volume). PCR was run as described above for 22 cycles. For the second round PCR, amplifications were performed in seven separate reactions. Each reaction contained: 2.5 μl of the appropriate first round PCR product, 1 μM of each primer, 1 U of *Taq* DNA polymerase, 0.1 mM of each dNTP and 2 mM MgSO_4_ (25 μl final volume). PCR was run as described above for 35 cycles. For TRPC3 and GFAP, the second round PCR required semi-nested and nested PCR, respectively. The sequences of internal primers are listed in Table 2.

PCR products were visualized on an ethidium bromide-stained agarose gel. Positive controls using 100 ng of total CA1 RNA were carried out in parallel for each single-cell amplification. GFAP primers were added as a control for glial RNA contamination and only cells that were GFAP negative were kept for data analysis. Controls for other contamination artefacts were performed by advancing a pipette into the slice without aspiration.

### Immunoprecipitation assays

Isolated hippocampus was homogenized (Polytron, 3 × 2 s, setting 4) (Glenn Mills, Clifton, NJ, USA) in ice-cold buffer containing: phosphate buffer saline (PBS), 1% Triton X-100, 1 mM NaF, 1 mM Na_3_VO_4_ and protease inhibitors (Cocktail inhibitor set I; Calbiochem). Homogenate was centrifuged at 20000 *g* for 20 min at 4 °C and the resulting supernatant was assayed for protein concentration using BCA (Pierce, Rockford, IL, USA). One milligram of proteins was incubated with 2 μg of rabbit polyclonal anti-TRPC1 (Sigma) for 30 min at 4 °C. The specificity of the immunoprecipitation was controlled by reabsorption of the antibody with equivalent amount of the antigen peptide overnight at 4 °C before incubation with tissue homogenate. Immune complexes were precipitated with 50 μl of 50% protein-G sepharose (Sigma) slurry for 90 min at 4 °C. The beads were washed three times for 10 min in PBS-1% Triton X-100 and proteins were eluted by the addition of electrophoresis sample buffer and heated at 65 °C for 7 min. Western blot detection was as previously described [13] using rabbit polyclonal anti-mGluR1a (1/500; Upstate Biotechnology, Lake Placid, NY, USA) and HRP-conjugated anti-rabbit IgG (1/10000; Jackson Immunoresearch, West Grove, PA, USA).

HEK-293 cells were grown and transfected as described previously [42]. In brief, cells were cultured in 6-well dishes and transfected with 1 μg of pRK5-mGluR1a (from Dr. J.P. Pin, Montpellier, France), and 1 μg of pcDNA3.1-FLAG-TRPC1 (from Dr. C. Montell, Baltimore, USA) or pcDNA3.1-myc-TRPC4 (from Dr. L. Méry, Paris. France) constructs. Two days after transfection, cells were collected in lysis buffer containing: 50 mM Tris pH 7.4, 120 mM NaCl, 1% Triton X-100, 2 mM EDTA, 1 mM NaF, 1 mM Na_3_VO_4_ and protease inhibitors. One well of confluent cells were used for each immunoprecipitation assay. Cleared supernatants were incubated with 2 μg of anti-FLAG (Sigma) or anti-mGluR1a. The rest of the procedure was the same as described above. The specificity of the immunoprecipitation was controlled by omitting to transfect the construct coding the immunoprecipitated protein. Western blot detection was performed using rabbit polyclonal anti-mGluR1a or mouse monoclonal anti-myc (1/5000, Sigma) and HRP-conjugated anti-rabbit or anti-mouse IgGs.

### TRPC1 and mGluR1a double-label immunofluorescence

Eighteen to twenty-one days old rats were deeply anaesthetized with intra-peritoneal injection of sodium pentobarbital (MTC Pharmaceuticals, Cambridge, Ontario, Canada) and transcardially perfused with a fixative solution containing 4% paraformaldehyde in 0.1 M phosphate buffer (PB, pH 7.4). The brain was dissected, postfixed for 4 h at room temperature and cryoprotected overnight in 30% sucrose. Transverse sections (45 μm thickness) were cut on a freezing microtome (Leica SM 2000R, Germany). Free floating sections were permeabilized 15 min in PBS-0.2% Triton X-100 and blocked 1 h at room temperature in PBS containing 10% NGS and 0.1% Triton X-100. Sections were incubated 4 days at 4 °C in a mixture of mouse monoclonal anti-mGluR1a (1/100 BD BioSciences, Oakville, ON, Canada) and rabbit polyclonal anti-TRPC1 (1/250) or rabbit anti-TRPC4 (1/100; Alomone Labs, Jerusalem, Israel). Sections were incubated 1 h 30 min at room temperature in biotinylated goat anti-rabbit IgG (1/200, Jackson ImmunoReasearch) and overnight at 4 °C in a mixture of Streptavidin-Alexa Fluor 488 (1/200, Invitrogen) and Texas Red conjugated goat anti-mouse (1/200; Jackson ImmunoResearch). Sections were rinsed thoroughly between incubations. All antibodies were diluted in PBS containing 2% NGS and 0.1% Triton X-100 (0.01% NaN_3_ was added for primary antibodies). Sections were mounted in ProLong Gold antifade reagent (Invitrogen) and observed with a Zeiss LSM 510 confocal microscope system (Axioskop; Coherent, Santa Clara, CA, USA) using appropriate filters.

### Western blots

For siRNA efficacy assessment, slices were homogenized by sonication in 50 μl of homogenization buffer containing: 50 mM Tris pH 7.4, 120 mM NaCl, 1% Triton X-100, 0.5% sodium deoxycholate, 0.1% SDS, 2 mM EDTA, 1 mM NaF, 1 mM Na_3_VO_4_ and protease inhibitors. The homogenate was then centrifuged at 20000 *g* for 20 min and protein concentration in the supernatant was determined as described above. Fifty micrograms of proteins per condition were subjected to western blot analysis as previously described [13]. Membrane was horizontally cut (~ 60 kDa molecular weight) to separate higher and lower molecular weight proteins. Top membrane was probed with rabbit polyclonal anti-TRPC1 (1/200) while bottom membrane was probed with mouse monoclonal anti-actin (1/1000, Santa Cruz Biotechnology, Santa Cruz, CA). After immunodetection, top membrane was stripped and re-probed with anti-TRPC4 (1/1000; Alomone; Jerusalem, Israel). Immunoreactive bands were scanned with a desktop scanner and quantified with Quantity One software (Bio-Rad, Hercules, CA, USA). The optical density of TRPC1 or TRPC4 was normalized to actin. Slices transfected with TRPC1 or TRPC4 siRNA were compared with slices transfected with scrambled siRNA. Only slices with comparable level of transfection, based on EYFP expression, were kept for analysis.

### Statistics

Summary data are expressed as mean ± s.e.m. Statistical significance was determined using two-tailed Student’s *t* tests for 2 groups comparisons (paired and unpaired comparisons indicated in text), or ANOVA followed by Bonferroni pair-wise comparisons for multiple groups, as appropriate. Significance level was set at *p* < 0.05.

## Results

### Slow EPSCs in O/A INs are mediated by TRP channels and independent of PLC signalling

**Fig. 1:**
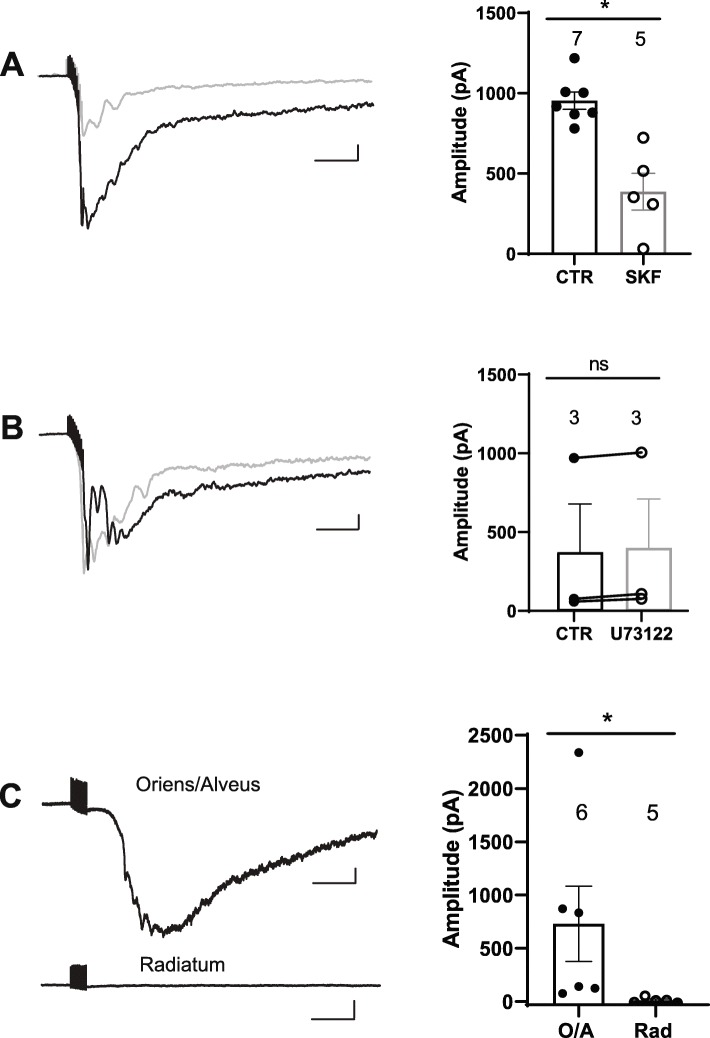
Slow EPSCs are mediated by TRP channels and independent of PLC in O/A INs, but absent in *stratum radiatum* interneurons. **a**. Left: representative traces of slow EPSCs elicited by HFS protocol in CA1 O/A INs of acute slices in control condition (black trace) and in presence of the TRP channel blocker SKF96365 (30 μM SKF, gray trace). Right: Summary bar graphs of peak EPSC amplitude for all cells in each condition. **b**. Left: representative slow EPSCs in O/A INs in acute slices in absence (black trace) and presence of PLC inhibitor (10 μM U73122, gray trace). Right: Summary bar graphs of peak EPSC amplitude for all cells in each condition. **c.** Left: Representative traces showing synaptic currents elicited by HFS in cultured hippocampal slices. Slow EPSCs were elicited in O/A INs (top), but were not elicited in *stratum radiatum* interneurons (bottom). Right: Summary bar graphs of peak EPSC amplitude for all cells in each condition. Numbers above bar graphs represent the number of cells in each group; *p < 0.05. Scale bars: 250 ms, 100 pA

Brief high-frequency stimulation (HFS) of hippocampal CA1 *stratum oriens* elicits in O/A INs a slow excitatory postsynaptic current (slow EPSC) mediated by mGluR1a and TRP channels [12]. Activation of group I mGluRs (mGluR1/5) by the selective agonist DHPG triggers membrane depolarization in CA1 O/A INs that is independent of PLC signalling [43]. However, the implication of PLC in slow EPSCs of O/A INs has not been investigated. Slow EPSCs were evoked in CA1 O/A INs of acute slices by HFS preceded by a brief depolarisation (to allow Ca^2+^ influx through voltage-gated Ca^2+^ channels) in the presence of TBOA (DL-*threo*-*b*-benzyloxyaspartic acid) (30 μM), a blocker of glutamate transporters (to enhance glutamate spillover to perisynaptic mGluR1a), as previously described [12]. In these conditions, HFS elicited slow EPSCs in O/A INs (Fig. 1 a). The amplitude of slow EPSCs was significantly reduced (41 ± 12% of control, *p* < 0.05, unpaired *t*-test) in the presence of the TRP channel antagonist SKF96365 (30 μM) and partially recovered after its washout (61 ± 12% of control). Slow EPSCs were not affected in the presence of the PLC inhibitor U73122 (10 μM) (Fig. 1 b, 109 ± 4% of control, *p* > 0.05, paired *t*-test), suggesting that slow EPSCS in O/A INs are mediated by TRP channels but may be independent of PLC signalling.

We then tested if slow EPSCs are present in CA1 O/A INs of hippocampal organotypic slices. In similar conditions as above, HFS evoked slow EPSCs in O/A INs in cultured slices (Fig. 1 c). Previous report showed that stimulation of mGluR1/5 in acute slices elicited only minimal membrane depolarisation and calcium transients in other CA1 interneurons located in *stratum radiatum/lacunosum-moleculare* [44]. Thus, we examined if slow EPSCs are elicited also in other interneurons in *stratum radiatum* in cultured slices. We found that HFS only elicited EPSCs with much smaller amplitude in *stratum radiatum* interneurons (Fig. 1 c; *p* < 0.05). Overall, these results suggest that slow EPSCs in CA1 O/A INs may be cell-type specific, mediated by TRP channel activation and independent of PLC signalling.

### TRPC mRNA expression in hippocampal CA1 region and in single O/A INs

**Fig. 2:**
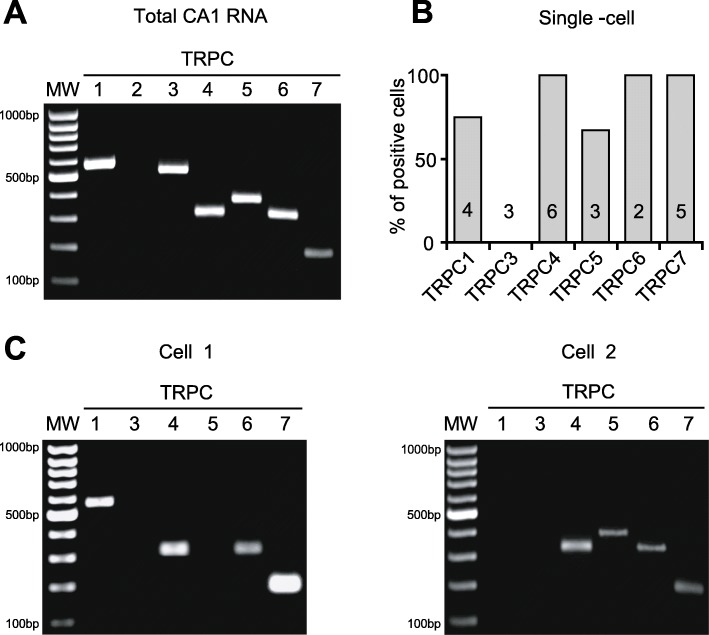
TRPC1, 3–7 mRNA expression in hippocampal CA1 region and TRPC1, 4–7 in individual O/A INs. **a.** Representative ethidium bromide-stained agarose gel of RT-PCR products for TRPC1–7 obtained from total CA1 mRNA (*n* = 3 separate experiments). **b.** Summary bar graph showing expression of TRPC1, 3, 4–7 mRNA detected by single-cell RT-PCR from CA1 O/A INs in which slow EPSCs were recorded. Numbers inside each bar represent the number of cells analyzed for respective TRPC member. **c.** Representative examples of gels of single-cell RT-PCR products obtained from two different cells. Lanes marked MW show the DNA molecular weight ladder

Among the TRP channel superfamily, TRPCs are a subfamily of channels that are highly expressed in brain and are associated with slow excitatory transmission [29, 32, 35, 45, 46]. Thus, we set out to examine the role of TRPCs in mGluR1a-mediated slow EPSCs in hippocampal O/A INs. So first, we determined mRNA expression of the seven TRPC members (TRPC1–7) in hippocampal CA1 region. Reverse transcriptase polymerase chain reaction (RT-PCR) detection of TRPC mRNAs was performed on total RNA isolated from microdissected CA1 region. We found that mRNAs for TRPC1, 3, 4, 5, 6 and 7 are expressed in CA1 region (Fig. 2 a).

Next we determined which TRPC mRNAs are expressed specifically in CA1 O/A INs. We performed simultaneous detection of the six TRPC mRNAs detected in CA1 region (TRPC1, 3, 4, 5, 6 and 7). After whole-cell recording of slow EPSCs in O/A INs, the cytoplasm was harvested into the patch pipette and multiplex single-cell RT-PCR was performed. Except for TRPC3, all other TRPC (TRPC1, 4, 5, 6 and 7) mRNAs present in CA1 region were detected in single O/A INs (Fig. 2 b). In additional whole cell recordings from O/A INs in which slow EPSCs was not tested (*n* = 3 cells), TRPC3 mRNA was not detected either. Different combinations of TRPC1, 4, 5, 6 and 7 mRNAs were observed in individual cells (ex. Fig. 2 c) but never all transcripts together. These results suggest that individual CA1 O/A INs express mRNA for TRPC1, 4, 5, 6 and 7, but not TRPC3.

### Molecular interaction of mGluR1a with TRPC1 and 4 in HEK-293 cells, and with TRPC1 in hippocampus

**Fig. 3:**
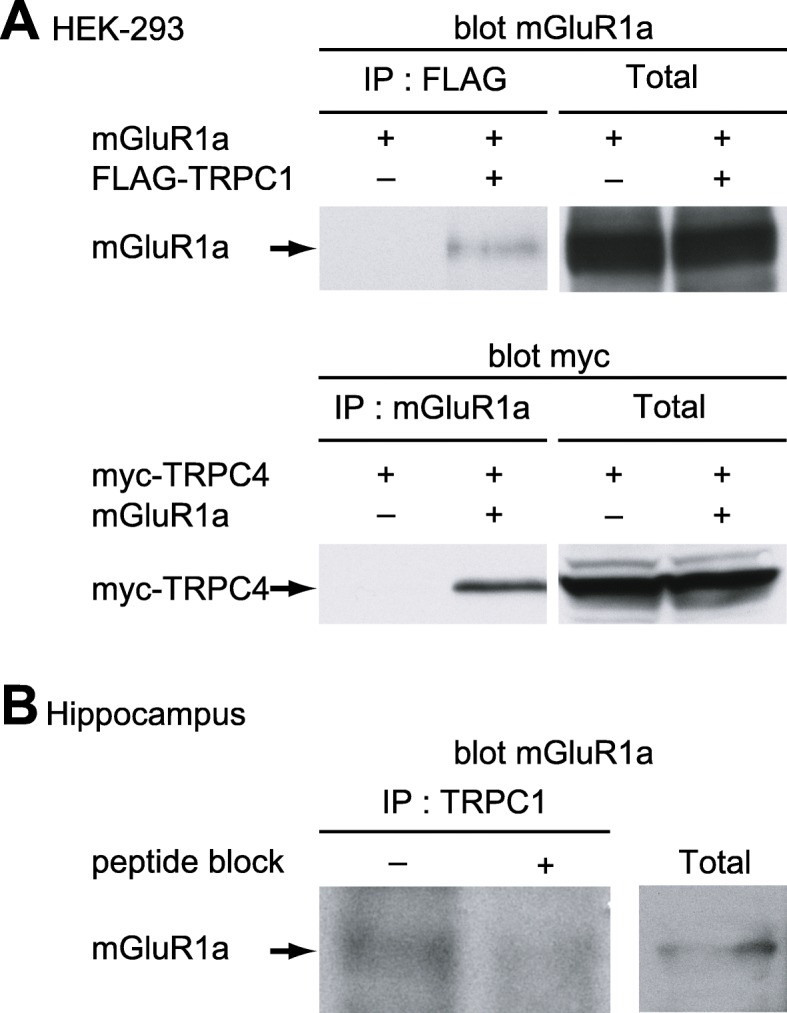
Molecular interaction of mGluR1a with TRPC1 and 4 in HEK-293 cells, and with TRPC1 in hippocampus. **a.** Top left: Cell extracts from HEK-293 cells transfected with mGluR1a alone, or with mGluR1a and FLAG-TRPC1, were immunoprecipitated with anti-FLAG and immunodetected by Western blot using anti-mGluR1a (*n* = 3 separate experiments). Top right: Immunodetection of mGluR1a from cell extracts. Bottom left: Cell extracts from HEK-293 cells transfected with myc-TRPC4 alone, or with myc-TRPC4 and mGluR1a, were immunoprecipitated with anti-mGluR1a and immunodetected using anti-myc (*n* = 3). Bottom right: Immunodetection of myc-TRPC4 from cell extracts. **b.** Left: Whole hippocampus tissue extract was immunoprecipitated with anti-TRPC1 in absence or presence of TRPC1 blocking peptide and immunodetected using anti-mGluR1a (*n* = 3). Right: Immunodetection of mGluR1a from whole hippocampus tissue extract

In cerebellum, TRPC1 and 3 are implicated in slow mGluR1-mediated EPSCs [29, 32, 33, 35], and mGluR1a and TRPC1 physically interact supporting an mGluR1a activation of TRPC1 [29]. Since we did not find evidence of TRPC3 expression in O/A INs, we focused on TRPC1 and TRPC4, another TRPC susceptible to act in slow synaptic transmission [29, 47]. To determine if mGluR1a associates with TRPC1 and TRPC4, we first performed co-immunoprecipitation assays in HEK-293 cells transiently transfected with mGluR1a and FLAG-tagged TRPC1, or myc-tagged TRPC4. Immunodetection of mGluR1a following TRPC1 immunoprecipitation with a FLAG antibody (Fig. 3 a), indicated a molecular interaction between mGluR1a and TRPC1. No signal was detected when TRPC1 was not transfected (data not shown). Similarly, immunodetection of myc-tagged TRPC4 after mGluR1a immunoprecipitation (Fig. 3 a), showed an interaction between mGluR1a and TRPC4. No signal was detected when mGluR1a was not transfected (data not shown).

Next, we examined whether these interactions occur in vivo by carrying out immunodetection of mGluR1a after immunoprecipitation of TRPC1 from hippocampal extracts (Fig. 3 b). We found that mGluR1a co-immunoprecipitated with TRPC1 and this was inhibited by preadsorption of the antibody with a blocking peptide (Fig. 3 b). Similar experiments with a TRPC4 antibody, failed to find mGluR1a co-immunoprecipitation with TRPC4 (data not shown). Our data suggest a molecular interaction of mGluR1a with TRPC1 and TRPC4 in recombinant system, but with TRPC1 in the hippocampus.

### TRPC1 and TRPC4 co-localize with mGluR1a in O/A INs

**Fig. 4:**
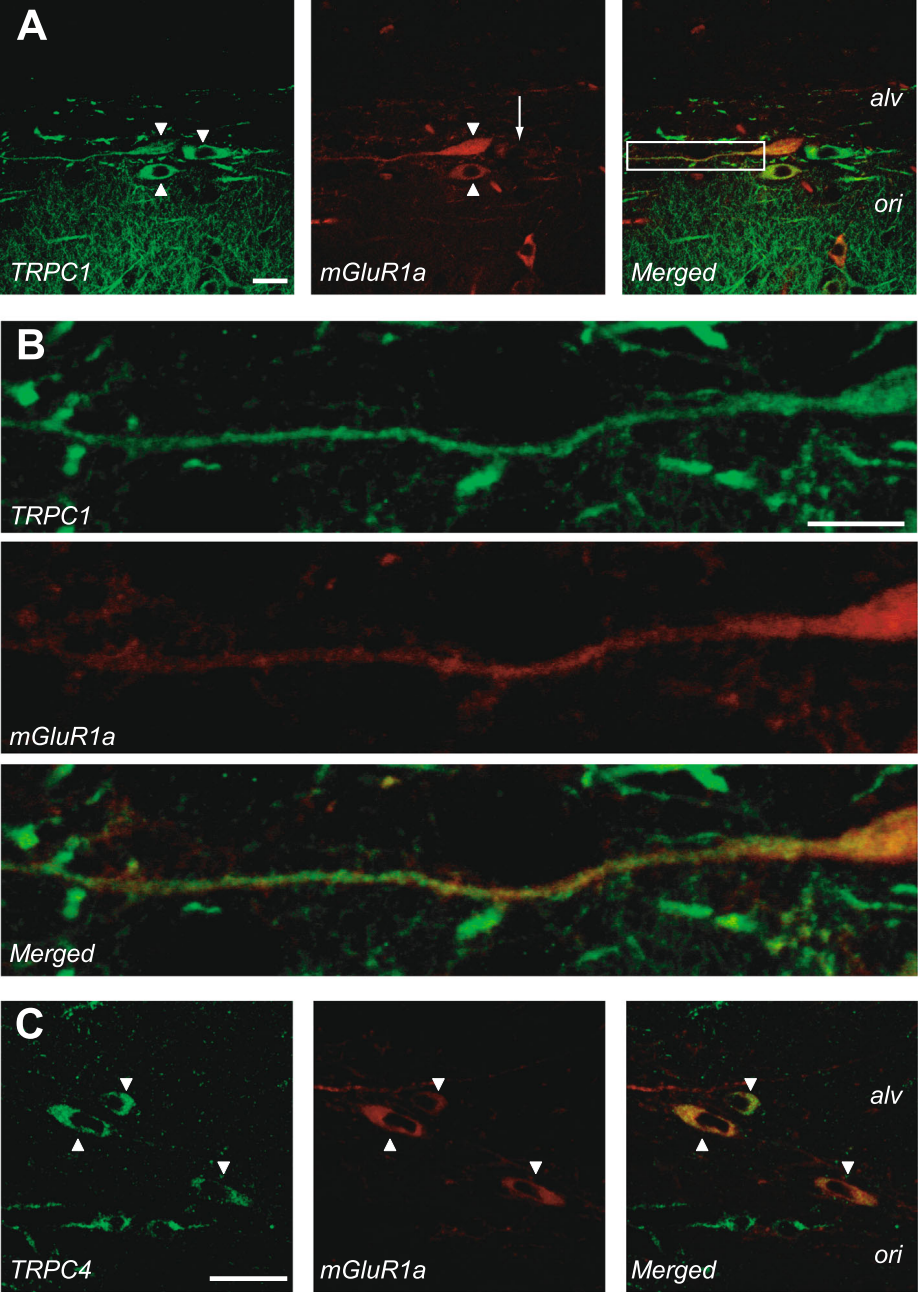
Co-localization of mGluR1a with TRPC1 and TRPC4 in O/A INs. **a.** Confocal images of double-label immunofluorescence of TRPC1 (green) and mGluR1a (red) in *stratum oriens/alveus* of the CA1 region. Left: TRPC1 antibody labelled the cell body and dendrites of three O/A interneurons (arrowheads), as well as other neuronal processes in *stratum oriens/alveus*. Middle: mGluR1a antibody also labelled cell bodies and proximal dendrites of the same interneurons. The cell body marked by an arrow showed less labelling than the two marked by arrowheads. Right: Merged images showing co-localization of TRPC1 and mGluR1a in cell bodies and proximal dendrites of O/A INs. **b.** Higher magnification confocal images of boxed region in A showing co-localization of TRPC1 and mGluR1a in a proximal dendrite of the O/A IN. **c.** Confocal images showing double-label of TRPC4 (green) and mGluR1a (red) in *stratum oriens/alveus* of the CA1 region. Left: TRPC4 antibody labelled cell bodies of O/A INs (arrowheads). Middle: mGluR1a antibody labelled the same cells. Right: Merged images showing co-localization of TRPC4 and mGluR1a. Scale bars: A and C, 25 μm; B, 10 μm

Next we examined the cellular co-localization of mGluR1a with TRPC1 or TRPC4 in hippocampal CA1 O/A INs using confocal microscopy and double-label immunofluorescence with antibodies against mGluR1a and either TRPC1 or TRPC4 (Fig. 4). TRPC1 antibody labelled neuronal processes in *stratum oriens/alveus* as well as cell body of interneurons, consistent with previous report [39]. In some cases, O/A interneuron dendrites were distinguishable from surrounding processes and these dendrites were positive for TRPC1 (Fig. 4 a-b). The mGluR1a antibody also labelled O/A INs and their dendrites (Fig. 4 a-b, middle), consistent with previous data [48]. Superimposition of the two labels showed co-localization of mGluR1a and TRPC1 in cell bodies and dendrites of O/A INs (Fig. 4 a-b). TRPC4 immunolabelling was also present in O/A INs but appeared limited to the cell body (Fig. 4 c). TRPC4 also co-localized with mGluR1a (Fig. 4C). These results show that TRPC1 has an overlapping distribution with mGluR1a in the cell body and dendrites of O/A INs while TRPC4 co-localization is predominant in the cell body.

### TRPC1, but not TRPC4, mediates slow EPSCs in O/A INs

**Fig. 5:**
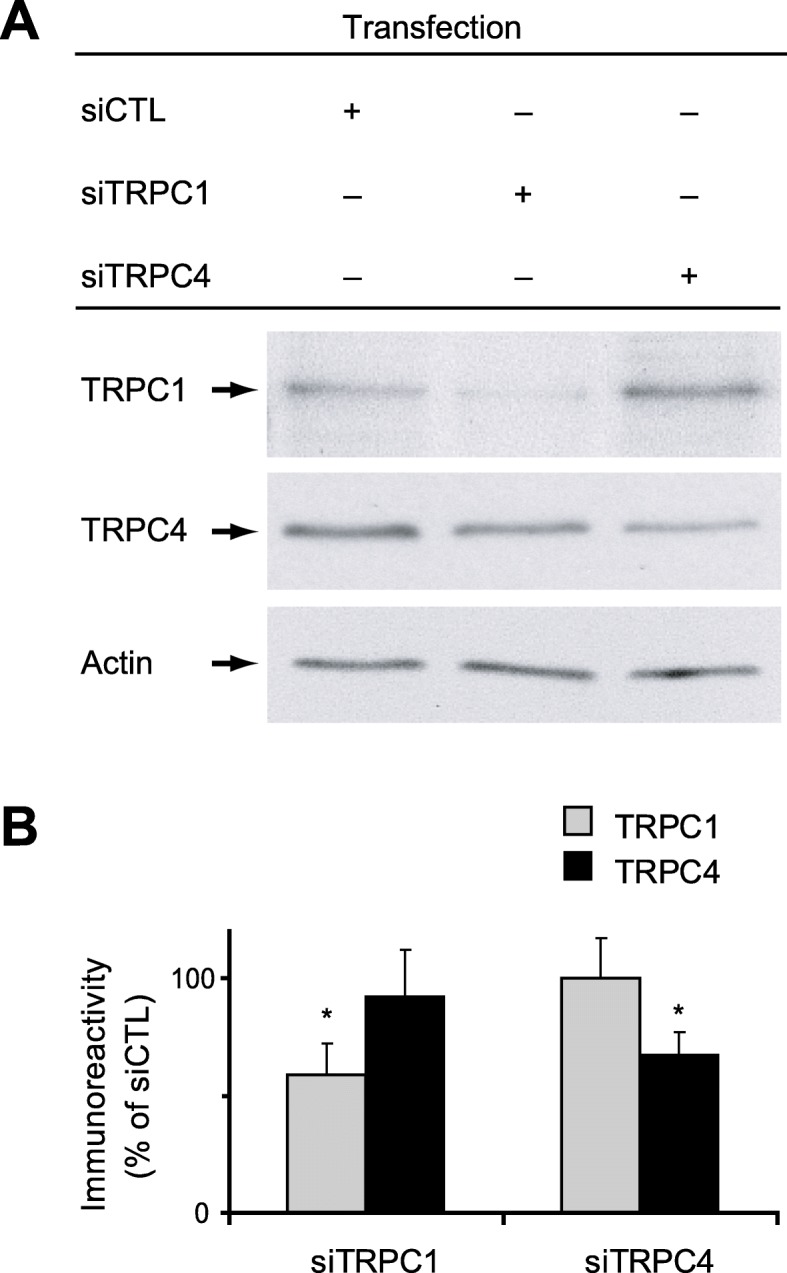
Selectivity of siRNA transfection targeting TRPC1 and TRPC4 expression in hippocampal organotypic slice cultures. **a.** Representative western blots of TRPC1 (top) and TRPC4 (middle) 48 h after biolistic transfection of hippocampal slice cultures with siCTL, siTRPC1 or siTRPC4, with Actin (bottom) used as loading control. **b.** Densitometric analysis of TRPC1 and TRPC4 expression normalized to actin value. Values in slices transfected with siTRPC1 or siTRPC4 were expressed relative to slices transfected with siCTL. Results are expressed as mean ± s.e.m. from at least 3 independent experiments. (*p < 0.05)

Next we examined whether TRPC1 or TRPC4 contributes to mGluR1a-dependent slow EPSCs in CA1 O/A INs using biolistic transfection of small interfering RNA (siRNA) in cultured hippocampal slices. First, we validated the specificity and efficacy of siRNAs targeting TRPC1 and TRPC4 by western blot. Transfection of siRNA targeting TRPC1 (siTRPC1) (Fig. 5) reduced expression of TRPC1 relative to slices transfected with scrambled siRNA (siCTL) (59 ± 13% of control; *p* < 0.05), without affecting TRPC4 expression (92 ± 20% of control; *p* > 0.05; *n* = 3). Conversely, transfection of siRNA targeting TRPC4 (siTRPC4) reduced TRPC4 expression (67 ± 10% of control; *p* < 0.05), but not TRPC1 (100 ± 17% of control; *p* > 0.05). Since mostly the superficial parts of slices are transfected with biolistic transfection in our conditions [49] and entire slices are used for western blot, the efficacy of siRNA is probably underestimated in our experiments. Nonetheless, our results confirm the specific protein knockdown of the respective target of siRNAs.

**Fig. 6:**
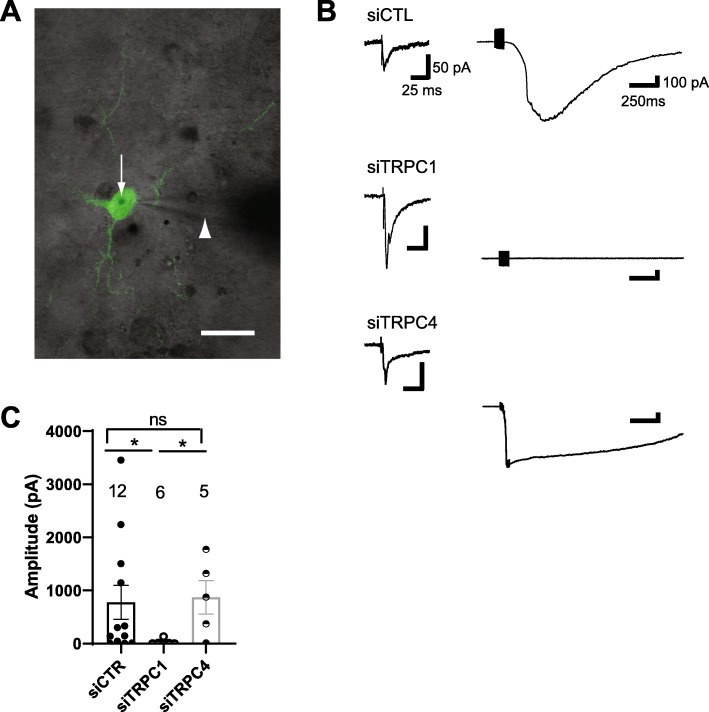
Selective knockdown of TRPC1, but not TRPC4, reduces slow EPSCs in O/A INs. **a**. Merged fluorescence and differential infrared contrast microscopy images showing a EYFP-expressing O/A IN. The gold particle from transfection is marked by an arrow and the patch pipette by an arrowhead. Scale bar: 50 μm. **b.** Representative recordings from EYFP-expressing O/A IN transfected with scrambled siRNA (siCTL; top), siRNA targeting TRPC1 (siTRPC1; middle), or siRNA targeting TRPC4 (siTRPC4; bottom). Left traces: Ionotropic glutamate receptor-mediated EPSCs evoked by a single stimulus. Right traces: slow EPSCs in the same cells. Calibration bar values are identical for the three groups) **c.** Summary bar graphs of amplitude of slow EPSCs for all cells. Numbers above bar graphs represent the number of cells in each group. (*p < 0.05)

Next, we co-transfected cultured slices with siRNA and EYFP, and used whole cell recordings from EYFP-expressing O/A INs to determine the role of TRPC1 and 4 in slow EPSCs (Fig. 6). In O/A INs transfected with siTRPC1, the amplitude of slow EPSCs was drastically reduced compared to EPSCs in O/A cells transfected with siCTL (Fig. 6 b-c; 4 ± 3% of control, *p* < 0.05). In contrast, in O/A INs transfected with TRPC4 siRNA slow EPSCs were not affected (Fig. B-C; 112 ± 18% of control; *p* > 0.05). To rule out unspecific effect of siRNAs on glutamatergic synaptic transmission, we recorded in the same O/A INs fast ionotropic glutamate receptor-mediated EPSCs evoked by single stimulus before bath application of CNQX and AP-5. Transfection of siTRPC1, siTRPC4 or siCTL did not affect these fast EPSCs (Fig. 6 b, left traces). Our results indicate that TRPC1, but not TRPC4, mediates slow EPSCs in O/A INs.

## Discussion

In this work, we examined the role of TRPCs in slow excitatory synaptic transmission in hippocampal O/A interneurons. We found that O/A INs express mRNA for TRPC1, 4–7, and that TRPC1 was co-localized with mGluR1a in O/A IN dendrites. Moreover, siRNA knock-down of TRPC1, but not TRPC4, impaired slow EPSCs in O/A INs. Thus, TRPC1 is a necessary component of mGluR1a-mediated slow excitatory synaptic transmission in O/A INs, and, consequently, may be involved in induction of Hebbian LTP at these synapses.

### TRPC expression and association with mGluR1a

TRPC channels are widely expressed in brain and they are known to mediate slow inward mGluR-dependent currents in many cell types [12, 13, 29–33, 35, 50]. To date, few pharmacological tools allows blockade of specific members of this family [51, 52]. Thus, studies that look at the expression and molecular interactions of TRPC channels with mGluRs are useful for the identification of TRPC members involved in this form of synaptic transmission.

RT-PCR analysis of TRPC expression revealed that, except for TRPC2, all TRPC transcripts are present in the CA1 region of hippocampus. These results are consistent with previous studies in rodents [16, 17, 36, 38–41]. However, these studies mostly focused on pyramidal cells and none of them specifically examined TRPC expression in interneurons. Single-cell RT-PCR revealed that O/A INs express TRPC1, 4, 5, 6, and 7 mRNAs in various combinations. The fact that we were unable to detect TRPC3 suggests that this member of TRPC family is absent or less common in O/A INs.

Immunofluorescence labelling showed that TRPC1 protein is expressed in interneurons of *stratum oriens/alveus*, consistent with previous report [39]. Our results show more precisely that TRPC1 is present in the cell body and dendrites of O/A INs where it co-localizes with mGluR1a. Such overlapping localization was also shown in midbrain dopamine neurons [34] and in auditory midbrain neurons [53], as well as in cerebellar Purkinje cells that display mGluR1a-mediated EPSCs [29]. Moreover, previous study reports a physical interaction between TRPC1 and mGluR1a in recombinant systems and in cerebellum [29]. Here, we show that this interaction between these two proteins is also present in the hippocampus. Taken together, our findings indicate the presence of both mGluR1a and TRPC1 in somato-dendritic compartment of O/A INs, a co-localization consistent with their functional interaction underlying EPSCs.

O/A INs also express TRPC4 protein, as shown previously [39]. However, in contrast to TRPC1, its immunolocalization appears limited to O/A IN cell bodies, and no molecular interaction was observed between TRPC4 and mGluR1a in the hippocampus. These results are in agreement with our finding using siRNA knock-down that TRPC4 is not required for slow EPSCs in O/A INs. TRPC1 was previously shown to be the only TRPC member to interact with mGluR1a in CHO cells [29]. However, in our hands, TRPC4 associates with mGluR1a in HEK-293 cells. This discrepancy could be explained by the variable levels of expression in heterologous systems.

### TRPC1 is involved in slow EPSCs in O/A interneurons

Brief high frequency stimulation induces slow inward mGluR1a-mediated EPSCs in O/A INs [12]. Pharmacological activation of mGluR1a in O/A INs leads to dendritic Ca^2+^ currents via TRP channels [13]. In the present work, we demonstrated that slow EPSCs are specific to CA1 O/A INs and are absent in *stratum radiatum* interneurons, and that TRPC1 mediates the slow mGluR1a-mediated EPSCs in O/A interneurons. Our findings are consistent with previous evidence that TRPC1 is involved in mGluR1a-mediated EPSCs in cerebellar Purkinje cells and GABAergic molecular layer interneurons [29, 33].

Slow EPSCs in O/A INs showed considerable cell-to-cell variability (Fig. 1 a-c). Such variability across individual cells may be explained by the presence of multiple cell classes in that region. However, in the original paper describing these slow EPSCs, similar high variability in response magnitude was reported and intracellular labelling identified the recorded cells as O-LM cells [12]. Thus, the reason for this variability remains unclear but it may not necessarily be due to recording from multiple cell classes.

Functional TRPC channels are formed of four subunits [54] and different TRPCs co-assemble in vivo to form heterotetrameric channels with properties distinct from homomeric channels [17, 22]. TRPC4 and TRPC5 were found to associate with TRPC1 in brain synaptosomes [55] and in the hippocampus [22]. In pyramidal cells of the lateral amygdala, mixed mGluR1/5-mediated EPSCs involve both TRPC1 and TRPC5 [31]. In layer 5 of prefrontal cortex, agonist activation of mGluR1/5 receptors induces currents in neurons with strong expression of TRPC4 and TRPC5 mRNAs [40]. In the present work, despite the co-localization of TRPC4 and mGluR1a in CA1 O/A INs, siRNA knock-down of TRPC4 did not affect slow EPSCs in these cells, suggesting an absence of functional interaction between TRPC4 and TRPC1 during slow EPSCs in O/A INs. However, our results do not exclude the possible involvement of other TRPC members (TRPC5–7) expressed in O/A interneurons, in addition to TRPC1, in mediating slow excitatory synaptic transmission in these cells.

### mGluR1a activation of TRPC1

Mechanisms underlying mGluR1a activation of TRPCs are still largely unknown. Here, our evidence suggests that TRPC-mediated slow EPSCs may be independent of the PLC pathway classically linked to group I mGluRs. In cerebellar Purkinje cells and GABAergic interneurons, as in midbrain dopamine neurons, mGluR1a-mediated EPSCs are also PLC-independent [33, 56–58] whereas in lateral amygdala mGluR1/5-mediated currents are PLC-dependent [31]. Thus, different mechanisms may link mGluR activation of TRPCs in a cell-specific manner.

Modulation of mGluR1a-induced currents and dendritic calcium transients by Src family tyrosine kinases and extracellular signal-regulated kinase (ERK) was reported in O/A INs [13]. Interestingly, in cerebellar Purkinje cells and GABAergic interneurons, as well as in midbrain dopamine neurons, PLC-independent mGluR1a-mediated currents also show tyrosine kinase modulation [33, 57, 58]. Thus, it will be interesting to investigate the role of these kinases in gating or modulating TRPC1 and slow EPSCs in O/A INs.

Stromal interacting molecule 1 (STIM1), a calcium sensor protein of the endoplasmic reticulum, was shown to gate TRPC1 channel under basal condition and upon receptor stimulation in different systems [59–62]. Activation of the channel was dependent on a lysine-rich region in the cytoplasmic domain of STIM1. Orai1 and caveolin were proposed to be partners with STIM1 in the activation of TRPC1, but the exact mechanisms of this gating are still under investigation [63, 64]. TRPC channels are also gated by conformational coupling with inositol-1,4,5-triphosphate receptors (IP_3_Rs) [65, 66] and ryanodine receptors (RyRs) [67]. This coupling is thought to be regulated by Homer proteins [68], a family of proteins that also interact with mGluR1a [69]. Since STIM1, Homer and TRPC have been shown, separately, to be implicated in mGluR dependent synaptic plasticity in Purkinje cell and hippocampus [13, 70, 71] it would be interesting to further investigate the role of these mechanisms in mGluR1a activation of TRPC1 in O/A INs.

### Functional implications

O/A INs express an mGluR1a-dependent Hebbian form of LTP at their excitatory synapses [5, 8, 9]. This LTP requires cytoplasmic calcium elevation [72]. In O/A INs, cytoplasmic calcium elevation following mGluR1a activation arises from two different sources: entry through plasma membrane TRP channels and release from intracellular stores [13]. The former is the main component of mGluR1a-mediated calcium transients and is required for LTP induction. Slow mGluR1a-mediated EPSCs are thus likely to participate in LTP induction by allowing calcium influx through TRP channels. This mechanism of induction seems to be cell type-specific, as interneurons of *stratum radiatum* and *lacunosum-moleculare*, which only display minimal mGluR1a-mediated EPSCs and calcium transients [44, 73], have different mechanisms of LTP induction [9, 74]. Here, we show that mGluR1a-mediated slow EPSCs in O/A INS are dependent on TRPC1, uncovering a new role for TRPC1 in O/A IN synaptic transmission, and likely crucial for Hebbian induction of LTP at these synapses.

Trpc1/4/5 triple knock-out mice show impaired hippocampal excitatory synaptic transmission, spatial working memory and cross-frequency coupling in hippocampal networks [22]. In addition, Trpc1^−/−^ mice have impaired spatial working memory and fear conditioning, as well as deficits in theta burst induced LTP and DHPG-induced LTD at Schaffer collateral-CA1 synapses [27]. These studies demonstrate the importance of TRPC1 and other TRPC members in regulating hippocampal synaptic network and behaviour. Given our evidence of the role of TRPC1 in excitatory synaptic transmission in inhibitory interneurons, it is interesting to speculate that some of these synaptic and behavioural effects may involve TRPCs in inhibitory neurons, possibly O/A interneurons. Hebbian mGluR1a-mediated LTP at excitatory synapses onto O/A INs, and more specifically onto interneurons that express somatostatin (SOM INs), was shown to upregulate persistently LTP magnitude at Schaffer collateral-CA1 pyramidal cell synapses via a disinhibition mechanism [5, 11, 75, 76]. Moreover, impairing mGluR1a-mediated LTP by interfering with mTORC1 function specifically in somatostatin interneurons impairs hippocampal spatial and contextual fear memory consolidation [11, 77, 78]. Thus since mGluR1a-mediated slow excitatory transmission in O/A INs is dependent on TRPC1 and that TRP channels blockade prevents LTP induction in O/A INs [13], TRPC1 function in interneurons may be important for hippocampal-dependent learning and memory.

In conclusion, we identified that TRPC1 mediates slow excitatory synaptic transmission at O/A INs synapses. Using patch-clamp recordings, we found that slow EPSCs in O/A INs are dependent on TRP channels but may be independent of phospholipase C. Using RT-PCR, we found that mRNA for TRPC 1, 3–7 was present in CA1 hippocampus, but using single-cell RT-PCR, we found expression of mRNA for TRPC 1, 4–7, but not TRPC3, in O/A INs. Using co-immunoprecipitation assays in HEK-293 cell expression system, we found that TRPC1 and TRPC4 interacted with mGluR1a. Co-immunoprecipitation in hippocampus showed that TRPC1 interacted with mGluR1a in vivo. Using immunofluorescence, we found that TRPC1 co-localized with mGluR1a in O/A IN dendrites, whereas TRPC4 localization appeared limited to O/A IN cell body. Finally, down-regulation of TRPC1, but not TRPC4, expression in O/A INs using small interfering RNAs prevented slow EPSCs, suggesting that TRPC1 is an obligatory TRPC subunit for these EPSCs. Our findings uncover a functional role of TRPC1 in mGluR1a-mediated slow excitatory synaptic transmission in O/A INs, suggesting an involvement of TRPC1 in induction of Hebbian LTP at these synapses.

## Data Availability

The datasets used and/or analyzed during the current study are available from the corresponding author on reasonable request.

## Abbreviations

EPSC: Excitatory postsynaptic current
HEK-293: Human embryonic kidney 293 cells
HFS: High frequency stimulation
LTP: Long Term Potentiation
mGluR1a: Metabotropic glutamate receptor 1a
O/A INs: *Oriens/Alveus* interneurons
O-LM: Oriens/lacunosum-moleculare
RT-PCR: Reverse transcription polymerase chain reaction
siRNA: Small interfering RNA
SOM INs: Somatostatin interneurons
TRP: Transient receptor potential
TRPC: Canonical transient receptor potential
